# Assessing the Influence of Physical Activity Upon the Experience Sampling Response Rate on Wrist-Worn Devices

**DOI:** 10.3390/ijerph182010593

**Published:** 2021-10-10

**Authors:** Alireza Khanshan, Pieter Van Gorp, Raoul Nuijten, Panos Markopoulos

**Affiliations:** 1 Department of Industrial Design, Eindhoven University of Technology, 5612 AZ Eindhoven, The Netherlands; p.markopoulos@tue.nl; 2 Department of Industrial Engineering, Eindhoven University of Technology, 5612 AZ Eindhoven, The Netherlands; p.m.e.v.gorp@tue.nl (P.V.G.); r.c.y.nuijten@tue.nl (R.N.)

**Keywords:** experience sampling method, ecological momentary assessment, context sensing, response rate, compliance, personalization, smartwatch application, wearables, physical activity

## Abstract

The Experience Sampling Method (ESM) is gaining ground for collecting self-reported data from human participants during daily routines. An important methodological challenge is to sustain sufficient response rates, especially when studies last longer than a few days. An obvious strategy is to deliver the experiential questions on a device that study participants can access easily at different times and contexts (e.g., a smartwatch). However, responses may still be hampered if the prompts are delivered at an inconvenient moment. Advances in context sensing create new opportunities for improving the timing of ESM prompts. Specifically, we explore how physiological sensing on commodity-level smartwatches can be utilized in triggering ESM prompts. We have created Experiencer, a novel ESM smartwatch platform that allows studying different prompting strategies. We ran a controlled experiment (N=71) on Experiencer to study the strengths and weaknesses of two sampling regimes. One group (N=34) received incoming notifications while resting (e.g., sedentary), and another group (N=37) received similar notifications while being active (e.g., running). We hypothesized that response rates would be higher when experiential questions are delivered during lower levels of physical activity. Contrary to our hypothesis, the response rates were found significantly higher in the active group, which demonstrates the relevance of studying dynamic forms of experience sampling that leverage better context-sensitive sampling regimes. Future research will seek to identify more refined strategies for context-sensitive ESM using smartwatches and further develop mechanisms that will enable researchers to easily adapt their prompting strategy to different contextual factors.

## 1. Introduction

In this study, we aim to motivate the relevance of adapting the time of delivering experiential questions during experience sampling studies. Specifically, we assessed the influence of physical activity on response rates during an experience sampling study that utilized commodity-level smartwatches. However, firstly, we introduce what the experience sampling method is and why finding the opportune moment of delivering the questions is crucial. The experience sampling method (ESM) was developed to collect data about behaviors, thoughts, or feelings in day-to-day activities in scientific studies involving human participants, addressing some of the shortcomings that characterize diary studies and retrospective surveys [1]. In these studies, participants are engaged for sustained periods of time during which they can be prompted at several moments of a day to provide a self-report regarding their emotions, thoughts, or experiences. Although ESM enables detailed examination of the phenomena under investigation [2], the compliance of participants in such studies is a long-standing challenge, hampering the effectiveness of the method [3]. Compliance to ESM studies is characterized by the volunteering rate (i.e., whether people accepts to participate in a study), the delay of a response (i.e., the time elapsing between a signal and the participant’s response), the amount of information presented, and the response rate. Compliance may be affected by response fatigue [4,5,6] caused by changes in motivation [7], attachment of participants to the outcome of the study [2], technical difficulties [8], and the extent to which participants experience the prompts as intrusive [3]. Earlier research already identified a variety of strategies for improving compliance that takes into account various factors including the age of participants, their education level, the timing of the prompt, the amount of information requested, and the weekly schedule of participants [9,10,11,12]. In this study, we focus on the response rate of participants, and we look for ways to improve it by tuning the timing of the prompt through utilizing contemporary wearable technology.

Advances in mobile and internet technologies enabled several innovations that aimed at improving the effectiveness of ESM protocols in general, and specifically, response rates. For example, Intille et al. proposed the use of response contingent sampling, manual specification of query times, flexible recurrence patterns (by weeks, days, hours, minutes), and bounded randomization (max/min times to next query) [13]. Hsieh et al. proposed the use of visualizations to increase compliance [14]. Another study showed that higher response rates can be achieved when participants can be allowed to specify the timing of the daily sampling [15]. Furthermore, the omnipresence of mobile devices, such as smartphones, created new opportunities for researchers to develop ESM software applications compatible with mobile devices [16]. By leveraging the capabilities of such devices, like detecting smartphone unlock [17,18,19,20], changes in location, using the microphone to detect silence or noise, and tracking calendar events, it is possible to detect sampling moments that are more convenient for the participants [21].

Modern ESM software solutions benefit researchers with their dynamic nature, giving them the flexibility to adjust study parameters such as notification schedules or incorporating complex logic in the questionnaires [22]. Markedly, the influence of different notification schedules, be it signal-contingent (random), interval-contingent or event-contingent is shown on the response accuracy and recall of the participants [23]. Arguably, choosing opportune moments to prompt (also known as a beep) participants may potentially decrease response fatigue, resulting in fewer dropouts, or even increase response quality.

In the past two decades, smartphones have accelerated the development of smarter ESM solutions that can run on study participants’ own devices which they carry anyway, rather than having to provide them with an extra dedicated device (as in early solutions using personal digital assistants like the Palm Pilot [24,25]). However, there can be numerous situations when individuals do not have their smartphones at hand. Furthermore, while recent smartphones support advanced physiological measurements (like electrocardiograms), such measurements can be obtrusive requiring participants to interrupt their activities, e.g., to put a finger on a specific part of the phone. In recent years, smartwatches have emerged as commodity devices that support less obtrusive and continuous sensing. Some smartwatches also enable the execution of third-party software and offer sufficient screen estate to support custom user interactions (e.g., Samsung Galaxy, and Apple Watch). These elements suggest the potential of supporting ESM through custom smartwatch applications. Additionally, these devices that are wrist-worn, easy to carry, and rich with sensors could be leveraged to facilitate reachability as well as understanding human behavior. More specifically, in the context of mHealth, novel means could be developed to tackle nowadays health problems (e.g., noncommunicative diseases, unexpected behavior during pandemics, inactivity among the populations, etc.). Accordingly, a promising direction is the development of ESM on wrist-worn devices. Via developing our custom ESM platform, Experiencer, we studied a dynamic form of experience sampling on smartwatches which makes use of sensors embedded in the smartwatch for choosing appropriate moments to sample user experiences. To demonstrate the potential of this approach we compared the compliance between (1) a group of ESM participants that was prompted when they were physically inactive (e.g., not moving or standing still), with (2) a group that was prompted while being physically active (i.e., walking, running, or doing any other activity such as householding). Assuming that people who are physically active are not as able to respond to prompts as those that are resting, we expected that the active group would have a lower response rate. Contrary to our expectations, active participants were more responsive. This result demonstrates the relevance of context-adaptive experience sampling.

The rest of the paper comprises the related works, materials and methods, results, discussion, and conclusions sections. We briefly review notable relevant works illustrating the use of commercially available wearable devices for experience sampling, their potentials, and limitations in the related work section. In the methods section, we introduce Experiencer, our prototype, and its distinctive features that benefited our ESM study and the study design. The results section includes the analysis of response rates during the study period. In the discussion section, we highlight our challenges, the limitations of our study, and we suggest interesting directions for future work and conclude by synthesizing and summarizing the insights gained with this study.

## 2. Related Work

The ubiquity of smartphones, along with their rich functionality, led to their widespread use in the context of ESM [26,27]. Advantages of in situ data collection using smartphones such as timestamping compared to that of conventional media like paper diaries [28] made them a favored choice for ESM studies and encouraged developments of ESM software solutions (e.g., [29,30,31,32]). Even though these technology-packed devices equipped with complex software help studies in many ways, the compliance of participants to long-running studies remains low [33]. In other words, response rates decline over time, especially when participants are required to respond frequently (e.g., 8 times a day) [34]. Choosing the opportune moments to prompt participants while adhering to the overall study protocol can be a way to mitigate these challenges. Numerous works examined how to do this by leveraging the capabilities of smartphones to gather contextual information [18,35,36,37,38].

Besides smartphones, newer mobile devices such as wearables offer new opportunities to support experience sampling studies [27,39,40]. Compared to smartphones, wearables can be more comfortable [41], with quicker accessibility [42], and can provide tactile feedback effectively as they are worn against the user’s body [40]. Researchers have argued that among wearables, smartwatches provide higher ecological validity [43], and are more appreciated by study participants compared to more bulky and sensor intensive wearables [44] such as Actiwatch or Shimmer. These devices provide dedicated sensing, but do not support functionalities that users typically need (e.g., reading email, receiving notifications, etc.). Hence, they are deemed as additional burden. Although, mainstream smartwatches have less accurate sensors compared to high-end sensor-focused wearables, their performance in recognition of physiological signals is acceptable for context sensing (such as physical activity [45], and emotion recognition [46]). Such commodity devices are promising to scale up to large ESM studies with hundreds of participants, without excessive budget requirements. To the best of our knowledge, recent studies with high-end sensory devices typically have sample sizes with less than 50 participants (N=19, M=20.15, SD=10.61) due to natural budget constraints. In addition to their usage in the ESM context, these devices are emerging as convenient and applicable tools for data collection and intervention in other domains such as in health (see [47,48,49,50]) and in cyber security (see [44,51]) as well.

Researchers begun to explore the use of wearable devices (e.g., smartwatches) for beeping, delivering questionnaires, and context sensing. These devices suffer from battery-life constraints and limited screen estate, which reduces usability and limits the possibility of both user input and content presentation [52,53]. Regardless, earlier studies showed that the time between an incoming notification (beep) and initial user interaction is significantly shorter with wearables compared to that of smartphones [54]. Additionally, as technology advances, newer generations of smartwatches provide improved usability and battery life, and improved content presentation, which makes them more attractive as a platform for supporting ESM.

## 3. Materials and Methods

To demonstrate how smartwatch sensors can be utilized in ESM studies and, more generally, to demonstrate the relevance of context-sensitive prompting in ESM, we set out to compare two sampling strategies that make use of physical activity sensors embedded in smartwatches. We assumed that participants will be more responsive during lower levels of physical activity. More specifically, we hypothesized that the response rate would be higher for beeps received when ESM participants are not moving compared to when they are physically more active. While many contextual factors may play a role in whether a participant will respond to an experience sampling beep (such as social context, or the tasks they are engaged in), we assumed that walking, running, or doing any other activity such as householding would impede user’s ability to respond and this would be reflected in response rates. The rest of this section introduces our prototyped software, study design choices, and data analysis steps.

### 3.1. Materials

**Figure 1:**
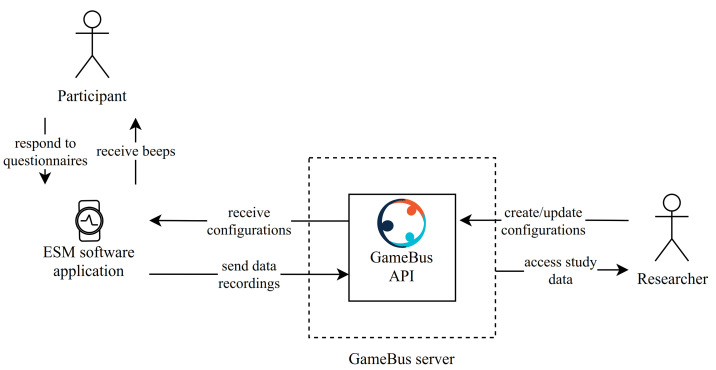
Overview of Experiencer and its integration with GameBus.

**Figure 2:**
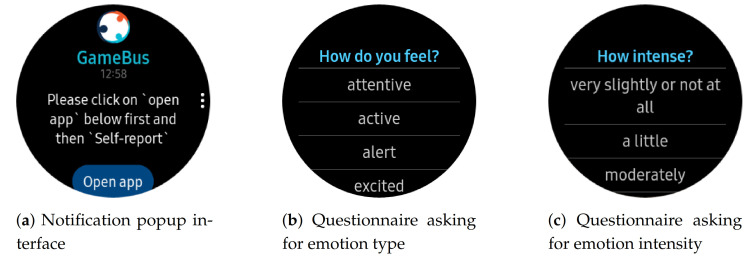
User interfaces of main interactions on ESM software application.

**Software**  We created *Experiencer* [55], a GDPR-compliant ESM platform. The software is implemented in JavaScript, using Web API of Tizen OS suitable for Samsung smartwatches. In our experiments, we used the Samsung Galaxy Watch Active 2 devices. To ensure seamless data collection, our prototype is integrated with *GameBus* [56] (an mHealth platform developed for supporting the design, implementation, and evaluation of various health promotion campaigns [57,58]) (Figure 1).
  *Experiencer* was designed to support (1) dynamic configurabilty that facilities researchers with on-the-fly adjustments applied to the ESM parameters. (2) stand-alone operation to collect data in situ, and syncing the data upon detecting reliable network connectivity. (3) a user interface compliant with wearable usability standards so that participants can easily answer the questionnaires on the smartwatch screen (Figure 2).

### 3.2. Methods

#### 3.2.1. Study Design

**Recruitment**  Our study was conducted in the context of the TU/e Samen Gezond program, an online program designed to promote healthy activities for the students and staff members of the Eindhoven University of Technology. During the program, participants received a set of healthy suggestions in a web application and were rewarded points in return for acting upon those suggestions. To enhance the experience of participants in the lifestyle program (by providing a steps tracker built on top of our ESM application), they also received Samsung Galaxy Watch Active 2 equipped with our prototyped ESM application.
**Duration**  The duration of the study was 5 weeks, which is as long as the TU/e Samen Gezond program lasted.
**Number of participants**  Constrained by the number of available smartwatches at the time of the study, and the recruitment process described, we could ultimately recruit N=71 participants.
**Treatment groups**  The participants were randomly assigned to two treatment groups which we called ’resting’ and ’active’: Half were assigned to the resting group who received beeps while not moving, and the other half to the active group who received beeps while being physically active (e.g., walking). Due to some early dropouts, ultimately the active group consisted N=37 and the resting group N=34 participants.
**Compensation**  Depending on the allocated treatment group in the TU/e Samen Gezond program, participants could be rewarded with a giveaway voucher of €25 in exchange for their points. Note that the participants were not rewarded for wearing the smartwatch neither for any other interactions with it (e.g., checking the smartwatch for notifications, replying to the questions they received, etc.). Rather they were rewarded for doing healthy activities that they could register via a separate web application dedicated to the TU/e Samen Gezond program or via unobtrusive sensing by the smartwatch.
**Schedule**  Following our hypothesis, the schedule of choice was event-contingent. The monitored event was the level of physical activity. As soon as a physical activity event was detected via our prototype, a beep was delivered to the participant’s smartwatch. The beeps were administered depending on the type of physical activity (e.g., walking, running, not moving), the treatment group a participant was in, and the defined inter notification time.
**Inquiry limit**  In our study, being event-contingent, sensible limits could reduce burden. According to the literature, around 7 beeps per day may yield an optimal balance of recall and annoyance [59]. Since we instructed participants to wear the smartwatch when they were awake, assuming one wears the smartwatch ∼12 h per day, an internotification time of 105 min (1.75 h) would result in $\frac{12}{1.75}<7$ inquiries per day, compliant with the literature.
**Inter notification time**  This notion is defined as the time in-between two consecutive notifications. In our case, since the schedule was event-contingent, there might be a situation that one is rarely or frequently beeped based on their level of physical activity and their treatment group. As as we described above, to prevent overwhelming the participants, we set a 105 min internotification time.
**Notification expiry**  There are many heuristics and hypotheses in the literature depending on different scenarios to determine notification expiry time (or lifetime) such as 5-min [60] or 3-min [61]. In this study, the notifications remained in the notifications area of the smartwatch, unless a participant cleared it, or the next beep from our prototyped ESM software arrived (our beeps did not stack up). This could also act as a reminder to the participant in case of an occasional visit to the notification area.
**Questionnaire**  To assess the impact of the event contingent strategy upon response rates, we chose to survey user emotions which is a typical case of ESM applications. Furthermore, we were motivated by earlier research that aims to infer emotions from wearable sensors (see [37,46,62]). Thus, at sampling moments, participants were requested to complete the Positive and Negative Affect Schedule (PANAS), which is a standard scale that consists of different words that describe feelings and emotions [63].

#### 3.2.2. Data Analysis and Cleaning

**Physical activity recognition**  To detect the physical activity levels of participants, we utilized the built-in Samsung pedometer API that applies its proprietary algorithm for physical activity detection. We adopted such an API to capture changes in physical activity in real-time and to manage sending beeps based on the physical activity levels of our participants across the active and resting treatment groups. More specifically, the pedometer API of the smartwatch is able to detect and distinguish *not moving*, *walking*, and *running* activities [64]. In the case that the algorithm fails to categorize a physical activity, it marks it as *unknown*. In our study, in the active group, the beeps were sent as soon as either *walking*, or *running* were detected and only if the internotification time was passed. In contrast, in the resting group, the beeps were sent when the *not moving* activity was detected in accordance with the inter notification time constraint. The internotification time was set to control the number of notifications sent to the participants. That is to avoid overwhelming the participants by sending a beep at any moment that the pedometer detects a physical activity. By setting such constraints, the participants received at most about 7 bees per day. Additionally, to capture a wider range of physical activities, we also leveraged detection of activities that fell under the *unknown* category. The details of such inclusion are described below in the Analysis section.
**Analysis**  The response rate is calculated as the ratio of the number of self-reports over the total number of received beeps. In the results section, we do so at the treatment group level both for the whole study period and on each week:
  $$response\_rate_{G_{t}}=\frac{|self\_reports_{G_{t}}|}{|beeps_{G_{t}}|}$$
  where *G* refers to a collection of participants containing either all members of a treatment group or a single participant. Also, the time window is referred to as *t*.
  The built-in physical activity monitor API of our smartwatch could detect walking, running, and not-moving activities. Additionally, to capture other physical activities (such as householding) we also enabled the detection of the built-in *unknown* physical activity [64]. By doing so, we were able to capture a wider range of physical activities (other than just walking and running) in line with our methodological decisions. The *unknown* event includes a spectrum of physical activities from subtle to vigorous and is triggered whenever the built-in activity monitor in the smartwatch fails to categorize a physical activity into either not moving, walking, or running. The *unknown* event may be detected both in lower (resting) or higher (active) levels of physical activity. Accordingly, we also checked the *speed* property of *unknown* events so that beeps were only delivered at intended levels of physical activity (e.g., for a participant in the active group, if an *unknown* activity of *high speed* were detected, a beep could be delivered).
**Cleaning**  The gathered data consisted of beep-related information, self-reports, and sensor data. The beep-related information consisted of timestamps of when a beep was received and when a beep was read. The self-report data included the timestamps of when the self-report was submitted, and the selected emotion from the PANAS scale along with its corresponding intensity (the different intensity levels are “very slightly or not at all”, “a little”, “moderately”, “quite a bit”, or “extremely”). The sensor recordings included the physiological data monitored to detect interesting events. i.e., active and resting states.
  As discussed in previous sections, the internotification time and the inquiry limit were set to specific values congruent with the common strategies in the literature (see [3,65]). However, at the beginning of our experiment, a technical malfunction in our first version of the prototype caused some constraint violations concerning the inquiry limits and inter notification times. That led to receiving beeps sooner than the intended inter notification time and more than the inquiry limit. In other words, participants received more beeps than intended. Although the issue was fixed during the first week of the study, some noisy data was generated. To clean such noises, in our analysis, for each participant, we only considered the first 7 beeps that were delivered on each day. Having cleaned data, we tested our hypothesis by calculating and then comparing the response rates of our treatment groups.

## 4. Results

Our 71 participants were divided into two treatment groups (resting and active). N=34 in the group notified at rest, and N=37 in the group notified while active. Although efforts were made to balance sample sizes, the number of participants in each group was not the same due to some early dropouts. Based on our study parameters, a maximum number of 17,393 beeps were possible to be delivered (71 participants, maximum 7 beeps per day, in 5 weeks). In practice, a total of 10,709 beeps were administered in our study, compliant with the calculated maximum possible beeps.

### 4.1. Response Rate

**Figure 3:**
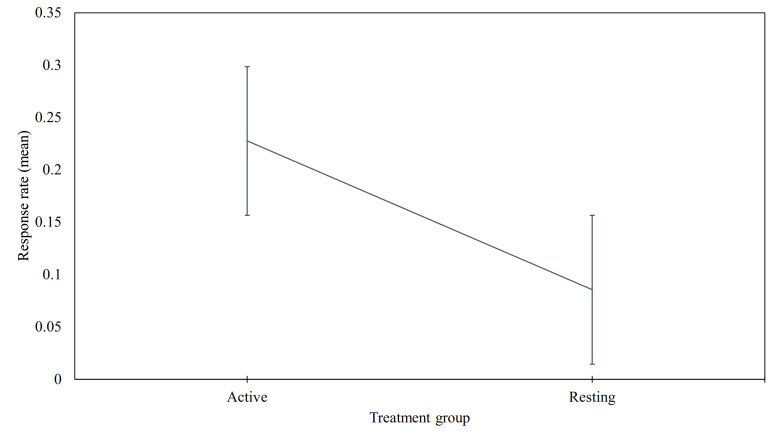
Comparison of response rates in treatment groups for whole study period.

We compared the response rates in each treatment group for the whole study period and also on a weekly basis. The normality of response rates obtained from our treatment groups was assessed. The Shapiro–Wilk test indicated that the data were not normally distributed, W=0.815,p=0.00004. According to the result of the normality test, and our intention to compare the means of two treatment groups, we adopted the Mann–Whitney U test (two-tailed, with alpha=0.05) to assess the difference. Based on our calculations we were able to reject our hypothesis. i.e., contrary to our expectations, not only was the descriptive mean response rate of the active group higher than the resting group, but also their difference was statistically significant. Our statistical test compared active group (M=0.227,SD=0.222) with resting group (M=0.085,SD=0.069), and resulted in p=0.001352, which supports its significance (Figure 3).

**Figure 4:**
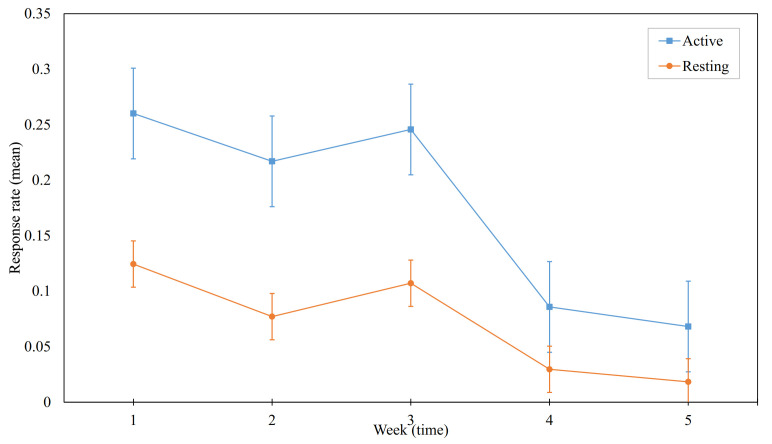
Comparison of response rates in treatment groups week-by-week.

Furthermore, we compared the differences in mean response rates of each group week-by-week. Based on our hypothesis, we expected higher response rates in the resting group, however, we observed otherwise. Meaning the descriptive means were in an unexpected direction for every week. However, we could only show statistical significance in the second and third weeks. Figure 4 illustrates the mean response rates of the active group in each week compared with that of the resting group.

By applying the Mann-Whitney U test, two-tailed, with alpha=0.05, we found significant difference in week 2 (i.e., p=0.0015), and week 3 (i.e., p=0.033).

### 4.2. Dropouts

**Figure 5:**
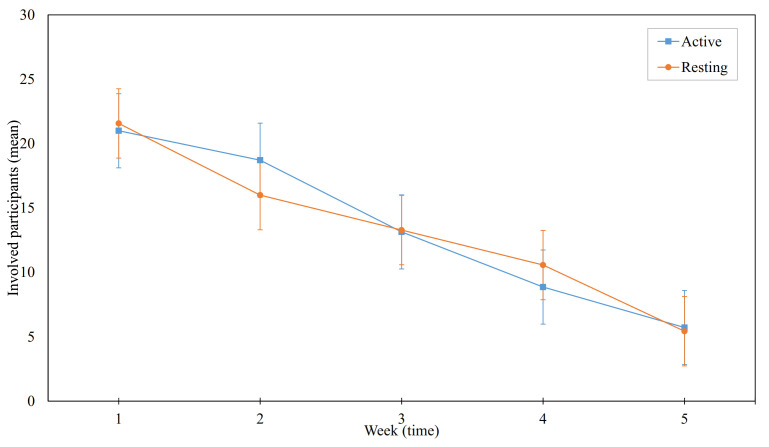
Comparison of participation in each treatment group.

As a complementary step, we also analyzed how involved our participants were throughout the study period and whether our event-contingent approach had any effect on the dropout rate. Based on our logs collected from the smartwatches, we accumulated the number of unique participants that used our prototype on each day. Ultimately, we failed to find any significant difference between the dropout rates of our treatment groups (M=13.5,SD=7.2 in the active group, and M=13.3,SD=6.2 in the resting group for the whole study period). In addition, we found a coefficient value of high degree (r=−0.8) in both groups with respect to time. That means the dropouts significantly increased as time passed. Figure 5 shows the mean of the total participating members in each treatment group every week. Nevertheless, we believe that our context-adaptive approach did retain a sufficient number of participants for roughly 4 weeks.

## 5. Discussion

Our research leveraged novel ESM software that exploits the capabilities of commercial wrist-worn devices for in situ context sensing. We prototyped Experiencer, our custom ESM platform compatible with smartwatches, and then successfully showed the effectiveness of an event-contingent experience sampling based on physical activity monitor data on response rate. However, a technical malfunction in our first delivered version of the prototype resulted in more beeps than intended, which possibly had an adverse impact on user experience and possibly caused dropouts. Our focus in this study was on finding the opportune moment to beep. To find such a moment, we modeled the user by a single piece of information, i.e., the level of physical activity. Including more contextual elements (e.g., current activity on the smartphone, prior responses, etc.) are interesting directions that could be studied in the future. Moreover, we considered response rate as the only determinant of compliance. Meanwhile, literature proposes other characteristics (e.g., delay of a response) to define compliance as well. Although the focus of our 5-week-long study was to find opportune interruption moments, long-term engagement and sustainability of such context-aware ESM regimes remain to be assessed for studies that last months or even years. Additionally, following the recent focus of the ESM literature on wearables, we only utilized smartwatches. Although the literature suggests that such devices will take over more conventional tools (e.g., smartphones) in the future and presents the superiority of smartwatches over smartphones in a variety of ESM studies, the inclusion of smartphones as well as smartwatches in future ESM studies would still enrich the obtained results.

Furthermore, information such as age and gender could provide more insights. Even though such information was asked during our discharge survey, they were not disclosed by most of our participants. The discharge survey also included a User Experience Questionnaire (UEQ) regarding the developed application and also the experience with the smartwatch itself. Such data could complement the analysis of response rates. However, the limited number of survey submissions made them inconclusive.

In addition, using a proprietary algorithm to detect physical activity imposes some limitations (e.g., inability to set a level of reference regarding the degree of physical activity to distinguish different activity types). On the other hand, devices that incorporate such algorithms and are tested by millions of retail customers indicate their robustness in the market and their potential in research settings. Respectively, context sensing via the ample sensors on the smartwatches requires further assessments by designing new studies that analyze the collected data against ground truth to understand the relationship between subjective self-reports and objective sensor data.

This study was part of the TU/e Samen Gezond program in which participants were given the chance to win a monetary reward in return for virtual points. The virtual points could be acquired if one followed the healthy suggestions throughout the campaign. We speculated that the participants who were more engaged and physically active in the program may be centered in one treatment group. However, we discovered that the participants who gained virtual points and monetary rewards were evenly distributed across our ‘resting’ and ‘active’ treatment groups. Thus, our treatment groups were not biased. Additionally, perceiving the participants’ mindset and goal setting regarding physical activity could add more value to the results. We identified and investigated the effect of personalized goal setting on engagement levels in our previous work [66]. Accordingly, the current study could be further complemented by evaluating individuals’ experiences and habits in terms of their day-to-day physical activity level upon the study inclusion stage.

## 6. Conclusions

In this paper, we investigated an event-contingent sampling schedule based on physical activity monitor data of commodity smartwatches to see how it influences the response rates in an experience sampling method (ESM) study. The experience sampling method addresses the issues of diary and retrospective study methods (such as retrospective bias), by distributing the sampling moments throughout the study period. However, ESM still suffers from declining response rates and increasing dropouts, especially in long-running studies. To specifically overcome the challenge of decreasing response rates, we prototyped our custom ESM platform, Experiencer, compatible with the Samsung smartwatches. To find the opportune moment to beep, we hypothesized that the level of physical activity at the moment of delivering beeps affects the response rates. More specifically, we expected that participants who were physically more active would have lower response rates and vice versa. Thus, we compared two treatment groups: resting against active. In the former, the beeps were delivered during lower levels of physical activity such as sedentary, and in the latter, the beeps were delivered in opposite situations. Contrary to our intuition, we rejected our hypothesis. i.e., the response rates in the active group were significantly higher than the ones in the resting group. Such results highlight the relevance of studying dynamic forms of experience sampling that leverage better context sensing and more intelligent sampling regimes, especially via commodity wearables such as smartwatches that are becoming widespread. Additionally, we discourage ESM tool builders from just implementing strategies based on intuition. On the other hand, we encourage scholars to conduct more fine-grained follow-up studies to better understand the optimal personalization settings concerning compliance in ESM studies.

## Data Availability

The data presented in this study are available on reasonable request from the corresponding author. The data are not publicly available to protect the privacy and anonymity of our participants.
